# Personal continuity of GP care and outpatient specialist visits in people with type 2 diabetes: A cross-sectional survey

**DOI:** 10.1371/journal.pone.0276054

**Published:** 2022-10-25

**Authors:** Anne Helen Hansen, May-Lill Johansen

**Affiliations:** 1 Faculty of Health Sciences, Department of Community Medicine, UiT The Arctic University of Norway *and* University Hospital of North Norway, Tromsø, Norway; 2 Faculty of Health Sciences, Department of Community Medicine, Research Unit for General Practice, UiT The Arctic University of Norway, Tromsø, Norway; Flinders University, AUSTRALIA

## Abstract

**Background:**

Continuity of care is particularly important for patients with chronic conditions, such as type 2 diabetes (T2D). Continuity is shown to reduce overall health service utilization among people with diabetes, however, evidence about how it relates to the utilization of outpatient specialist services in Norway is lacking. The aim of this study was to investigate continuity of GP care for people with T2D, and its association with the use of outpatient specialist health care services.

**Methods:**

We used e-mail questionnaire data obtained from members of The Norwegian Diabetes Association in 2018. Eligible for analyses were 494 respondents with T2D and at least one GP visit during the previous year. By descriptive statistics and logistic regressions, we studied usual provider continuity (UPC) and duration of the patient-GP relationship and associations of these measures with somatic outpatient specialist visits. Analyses were adjusted for gender, age, education, self-rated health, and diabetes duration.

**Results:**

Mean age was 62.6 years and mean UPC was 0.85 (CI 0.83–0.87). Two thirds of the sample (66.0%) had made all visits to the regular GP during the previous year (full continuity). Among these, 48.1% had made one or more specialist visits during the previous year, compared to 65.2% among those without full continuity. The probability of outpatient specialist visits was significantly lower among participants with full continuity, compared to those without full continuity (Odds Ratio 0.53, Confidence Interval 0.35–0.80). The probability of visiting outpatient specialist services was not associated with duration of the patient-GP relationship.

**Conclusions:**

We conclude that continuity of care, as measured by Usual Provider Continuity, is high and associated with reduced use of somatic outpatient specialist services in people with T2D in Norway. Continuity and its benefits will become increasingly important as the number of older people with diabetes and other chronic diseases increases.

## Introduction

### Diabetes is an increasing health challenge

Diabetes is an increasing health challenge worldwide, estimated to affect 8,8% of the adult population in the world [1]. The Norwegian prevalence is around 5% [2], estimating that around 270,000 are diagnosed with diabetes. Of these, around 247,000 have type 2 diabetes (T2D) [2]. More than 10% of the population over 80 years have T2D, and prevalence is increasing [2]. Most Norwegian patients do not reach the combined national treatment targets for prevention of complications [3, 4], and diabetes poses a considerable burden in terms of morbidity and mortality [5].

### Continuity in previous research

Continuity is a core value of general practice, and is often seen as the relationship between a single practitioner and a patient that extends beyond specific episodes of illness or disease.

Continuity of general practitioner (GP) care is associated with patient satisfaction [6], compliance [7], comprehensiveness of care [8], and enhanced receipt of preventive services [9]. Furthermore, it is associated with reduced service duplication [10], reduced use of complementary and alternative medical providers [11], emergency departments, specialist visits, hospitalisations [12–16], and reduced expenditures in hospital care [15, 16]. And most importantly; continuity is associated with reduced mortality [12, 17–19].

Continuity is believed to have few negative associations, but reduced rapid access to care, delayed diagnosis, and a loyalty and dependency that may harm the patient have been mentioned [9, 20].

### Continuity and chronic disease

Personal continuity of care is particularly important for patients with chronic medical conditions [21]. In diabetes populations in different health care systems around the world, continuity is suggested to reduce overall health service utilization [22], use of emergency departments [23], hospitalizations [22–24], medical costs [23, 25], health complications [22, 23, 25], and mortality [22, 24].

### Norwegian health care

Norwegian health care is based on universal insurance. The Regular General Practice (RGP) system was implemented in 2001, aiming to improve quality, accessibility, and continuity in general practice. Inhabitants are invited to choose their own individual GP, who mostly work in group practices. By 2022, each GP serves around 1050 inhabitants [26]. Routine diabetes follow-up is usually performed by the patient’s regular doctor without practice nurses involved. GPs themselves thus have a major role in managing T2D [27]. As a system, the Norwegian GP scheme gives strong incentives to personal continuity of care, however, in recent years the system has been increasingly challenged by heavy workloads and lack of recruitment [28, 29]. An increasing part of the Norwegian population do not have a real choice of GP, and according to The Norwegian Association of General Practitioners at least 235,000 residents (around 4,3% of the population) were without a GP by August 2022.

Access to Norwegian specialist care is considered good and is usually achieved by referral from the GP (the gatekeeper role). Norwegian health services are organized according to the Lowest Effective Care Level principle (LEON), meaning that health care should be provided at the lowest effective level of care. Good referral practice thus means that GPs are encouraged to refer patients who need assessment or treatment in specialist care, and not patients who may be as well or better served by primary health care services [30, 31].

### Relevance of the study

In Norway and many other countries, continuity is threatened by a lot of factors, such as increased migration, professional development, information technology, and demands for accessibility and plurality of services [14, 32, 33]. These are mostly positive changes in society that we do not want to combat. However, the current crisis in recruitment and stabilisation of general practitioners in Norway may hamper continuity, and the crisis increases the importance of research like the current project. Solid evidence about how continuity of GP care may impact the utilization of outpatient specialist services in patients with T2D in Norway is lacking.

### Aim

The aim of this study is to estimate the relational continuity of GP care for people with T2D by measuring the use of their regular GP versus other GPs, and the duration of the patient-GP relationship. Furthermore, we aim to investigate the association between these two relational continuity measures and the use of outpatient specialist health care services, adjusted for sociodemographic and health related patient characteristics.

## Methods

### Data

For this cross-sectional study we used questionnaire data from the DIAcare study [34] obtained by e-mail in 2018 from members of The Norwegian Diabetes Association. By 31.12.2017, the organization had 33,908 members, 53% women and 47% men. Around 70% of the members had T2D [35]. The Norwegian Centre for Research Data (NSD) Web Survey distributed the invitations to a randomly selected sample of 5,971 individuals (about 18% of all members).

The questionnaire (S1 Questionnaire) was developed from the research group’s experiences and from relevant questionnaires used in other published surveys [36]. We included questions about demographic and socio-economic characteristics, health status, specific questions about diabetes duration, control, severity, and treatment, and use of health care services. The questionnaire was reviewed and tested several times before data collection by two persons diagnosed with diabetes, and by experts from our research group.

Information about the study purpose and what participation would entail was distributed together with the invitation. A second invitation was sent to non-respondents 15 days after the initial request.

### Participants

**Fig 1:**
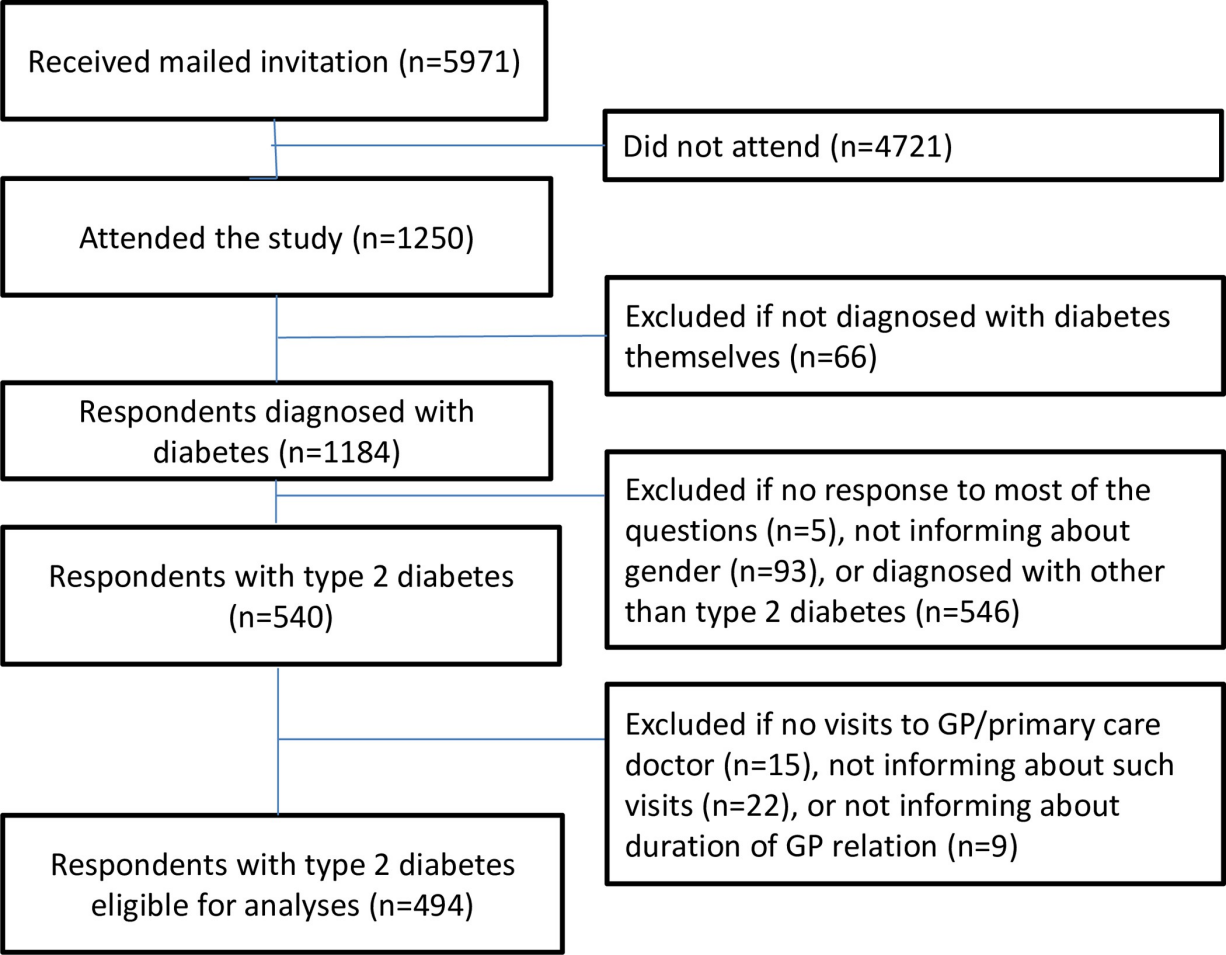
Flow chart of study population.

From the 1,250 participants, we excluded those who did not suffer from diabetes themselves (n = 66). This group consisted of 61 family members, 4 health personnel (2 overlapping), and 3 others. Further, we excluded those who did not respond to most of the questions (n = 5), those who did not inform about gender (n = 93), and those diagnosed with other diabetes types than T2D (n = 526). Since our focus in this study was personal continuity of GP care, we finally excluded participants who had not visited their GP during the previous 12 months (n = 15), or did not answer regarding GP visits (n = 22), or did not inform about duration of their GP relation (n = 9). The sample finally consisted of 494 respondents (Fig 1).

### Variables

The dependent variable was use of somatic specialist outpatient services once or more during the previous 12 months. Specialist service use refers to any somatic specialist clinic visit, regardless of clinical issue (not only endocrinologists/diabetologists).

The core independent variables for measuring continuity of care were Usual Provider Continuity (UPC) and duration of the patient-GP relationship (GP duration). UPC measures the proportion of all GP visits made to the regular GP, meaning that UPC equals one (1) if all primary care doctor visits are made to the regular GP [37]. Since we were interested in full continuity versus not full continuity according to this measure, we dichotomised this variable into UPC = 1 and UPC<1. Response options for duration of the patient-GP relationship were <1 year, 1–2 years, 3–4 years, and >4 years. Since most participants (361 respondents) reported more than 4 years with the same GP, we dichotomised this variable into 0–4 years and >4 years.

Adjustment independent variables were gender, age, education, self-rated health, and diabetes duration. We grouped age in 20-year intervals. The four education categories were labeled low (primary/part of secondary school), middle (completed secondary school), high (college/university < 4 years), and highest (college/university 4 years or more). Response options for self-rated health were excellent, good, fair, bad, and very bad. The bad and very bad categories were merged due to low numbers in the very bad category (4 respondents). Diabetes duration was grouped in 10-year intervals, however, participants with 30 years or more with the diagnosis were merged into one group.

### Analyses

Data were analyzed by descriptive statistics and logistic regressions. Correlations were tested with Spearman’s and Pearson’s correlation coefficients. We constructed one multivariate logistic regression model for each of the two core independent variables. The adjustment independent variables were introduced collectively into the models.

Due to a relatively low response rate, we compared respondents who did not respond initially but eventually consented with early respondents, assuming that late respondents were more similar to non-respondents [38]. This was done by stratifications, and by subsequently introducing the response time variable into the regression models. To ensure it did not influence on our results, we also performed similar analyses after excluding those who had made only one GP visit during the previous year.

We used 95% confidence intervals (CI) throughout the study. For the analyses we used Stata, version 17.0.

### Ethics

An adjoining project, using the same dataset, has been presented to the Regional Committee for Medical and Health Research Ethics (REK), which found that an application was not required according to the Norwegian Health Research Act (2015/1779/REK nord). All aspects of several adjoining projects using the same dataset have been approved by the Data Protection Officer (Personvernombudet) at the University Hospital of North-Norway (ref 2017/6579, ref 2018/5268, and ref 2019/3761). The data bureau NSD handled the data collecting process accordingly, and received no other information about the participants than the e-mail addresses. Written informed consent was obtained from the participants. Personal data were not identifiable to the researchers. All methods were carried out in accordance with the Declaration of Helsinki.

## Results

### Participation

In total 1250 persons aged 18–89 years attended the study, constituting an overall response rate of 21%. Due to more than 400 bounce backs from servers unable to deliver the invitation, and also experiences that membership registration and e-mail addresses might not be completely updated, we assume the real participation rate to be higher. After exclusions (Fig 1), 494 individuals with T2D who had visited their GP at least once during the previous year were eligible for analyses.

### Characteristics of the participants

Mean age of participants was 62.6 (CI 61.7–63.5) years, and median age 63 years. Most of the sample (73.1%) had been registered with the same GP for more than 4 years. The largest groups were men (62.3%), people in the two highest education groups (50.1%), and people with good/excellent self-rated health (63.9%). Duration of the patient-GP relationship was more than 4 years for 73.1% of the sample, and 66.0% had made all GP visits to their regular GP during the previous year. One or more specialist outpatient visits were made by 53.9% of the participants during the previous year (Table 1).

**Table 1:** Sample characteristics.

	Total sample n (%)	Early respondents n (%)	Late respondents n (%)	Respondents with UPC<1 n (%)	Respondents with GP duration 0–4 years n (%)
**Specialist visits**^**a**^ **(n = 482)**					
No visits	222 (46.1)	125 (42.8)	97 (51.0)	59 (34.8)	59 (45.7)
One or more visits	260 (53.9)	167 (57.2)	93 (49.0)	107(65.2)	70 (54.3)
**Usual Provider Continuity (UPC) (n = 494)**					
<1 (some visits to other than regular provider)	168 (34.0)	101 (33.9)	67 (34.2)	168 (100.0)	57 (42.9)
1 (all visits to regular provider)	326 (66.0)	197 (66.1)	129 (65.8)	0 (0.0)	76 (57.1)
**Duration of patient-GP relationship (n = 494)**					
<1 year	33 (6.7)	17 (5.7)	16 (8.2)	15 (8.9)	33 (24.8)
1–2 years	43 (8.7)	31 (10.4)	12 (6.1)	22 (13.1)	43 (32.3)
3–4 years	57 (11.5)	33 (11.1)	24 (12.2)	20 (11.9)	57 (42.9)
>4 years	361 (73.3)	217 (72.8)	144 (73.5)	111 (66.1)	0 (0.0)
**Gender (n = 494)**					
Female	186 (37.7)	104 (34.9)	82 (41.8)	74 (44.0)	53 (39.8)
Male	308 (62.3)	194 (65.1)	114 (58.2)	94 (56.0)	80 (60.2)
**Age (n = 494)**					
18–39	11 (2.2)	9 (3.0)	2 (1.0)	5 (3.0)	1 (0.8)
40–59	154 (31.2)	106 (35.6)	48 (24.5)	53 (31.5)	45 (33.8)
60 years and over	329 (66.6)	183 (61.4)	146 (74.5)	110 (65.5)	87 (65.4)
**Education**^**b**^ **(n = 492)**					
Low	62 (12.6)	35 (11.7)	27 (13.9)	16 (9.6)	19 (14.4)
Middle	159 (32.3)	94 (31.5)	65 (33.5)	54 (32.3)	43 (32.6)
High	144 (29.3)	93 (31.2)	51 (26.3)	48 (28.8)	38 (28.8)
Highest	127 (25.8)	76 (25.5)	51 (26.3)	49 (29.3)	32 (24.2)
**Self-rated health (n = 488)**					
Excellent	57 (11.7)	36 (12.2)	21 (10.9)	14 (8.5)	12 (9.1)
Good	255 (52.3)	142 (48.1)	113 (58.5)	84 (50.9)	72 (54.5)
Fair	130 (26.6)	84 (28.5)	46 (23.8)	49 (29.7)	36 (27.3)
Bad/very bad	46 (9.4)	33 (11.2)	13 (6.7)	18 (10.9)	12 (9.1)
**Diabetes duration (n = 492)**					
< 10 years	187 (38.0)	114 (38.4)	73 (37.4)	58 (33.9)	55 (41.4)
10–19 years	185 (37.6)	116 (39.1)	69 (35.4)	70 (41.7)	49 (36.8)
20–29 years	93 (18.9)	50 (16.8)	43 (22.1)	31 (18.5)	23 (17.3)
30 years and over	27 (5.5)	17 (5.7)	10 (5.1)	10 (5.9)	6 (4.5)

a: One or more specialist outpatient visits during the previous 12 months

b: Low (primary/part of secondary school), Middle (high school), High (college/university < 4 years), Highest (college/university 4 years or more)

Annual consultation rate was 5.08, and mean UPC was 0.85 (CI 0.83–0.87). In total, 21 of the participants had visited a GP only once. Two of these had consulted another than their regular GP.

Among participants with UPC = 1, 48.1% had made one or more specialist visit during the previous year, compared to 65.2% among those with UPC<1. Among participants with a GP duration of more than 4 years, 53.8% had made at least one specialist visit during the previous year, compared to 54.3% among those with a shorter GP duration.

The sample consisted of 298 early respondents (60.3%) and 196 late respondents (39.7%). The late respondents were older than the early respondents (74.5% vs 62.4% were 60 years and over), consisted of more women (41.8% vs 34.9%), had better self-reported health (69.4% vs 60.3% in good/excellent health), and had less visits to specialist services during the previous year (49.0% vs 57.2%) (Table 1).

### Associations of outpatient specialist visits and continuity measures

The probability of visiting outpatient specialist services was significantly lower among participants with all primary care visits to the regular GP, compared to those who had made at least one visit to other primary care doctors (OR 0.53, CI 0.35–0.80) (Table 2).

**Table 2:** Probability of one or more outpatient specialist visits according to Usual Provider Continuity (UPC) (N = 472).

	Outpatient specialist visits (yes/no)		
	OR	p	CI
**UPC** ^**a**^			
<1^b^	1.00		
1	**0.53**	**0.003**	**0.35–0.80**
**Gender**			
Female^b^	1.00		
Male	1.14	0.511	0.76–1.72
**Age**			
18–39 years^b^	1.00		
40–59 years	0.73	0.638	0.20–2.69
60 years and over	1.05	0.945	0.28–3.87
**Education** ^**c**^			
Low^b^	1.00		
Middle	1.22	0.536	0.65–2.30
High	**1.98**	**0.038**	**1.04–3.77**
Highest	1.75	0.097	0.90–3.38
**Self-rated health**			
Excellent^b^	1.00		
Good	1.47	0.224	0.79–2.75
Fair	**2.79**	**0.003**	**1.41–5.51**
Bad/very bad	**2.82**	**0.017**	**1.21–6.58**
**Diabetes duration**			
< 10 years^b^	1.00		
10–19 years	1.16	0.513	0.74–1.84
20–29 years	1.71	0.065	0.97–3.02
30 years and over	**2.99**	**0.033**	**1.09–8.18**

Statistically significant findings are marked in bold

OR odds ratio; CI confidence interval

a: UPC Usual Provider Continuity <1: some visits to other than regular provider, UPC 1: all visits to regular provider

b: Reference groups

c: Low (primary/part of secondary school), Middle (high school), High (college/university < 4 years), Highest (college/university 4 years or more)

The probability of visiting outpatient specialist services was not associated with duration of the patient-GP relationship (Table 3).

**Table 3:** Probability of outpatient specialist visits according to duration of the patient-GP relationship (N = 472).

	Outpatient specialist visits (yes/no)		
	OR	p	CI
**Duration of the patient-GP relationship**			
0–4 years ^a^	1.00		
>4 years	0.94	0.783	0.61–1.44
**Gender**			
Female^a^	1.00		
Male	1.06	0.765	0.71–1.58
**Age**			
18–39 years^a^	1.00		
40–59 years	0.68	0.566	0.19–2.49
60 years and over	0.96	0.952	0.26–3.50
**Education** ^b^			
Low^a^	1.00		
Middle	1.30	0.405	0.70–2.43
High	**2.07**	**0.025**	**1.10–3.93**
Highest	1.89	0.056	0.98–3.62
**Self-rated health**			
Excellent^a^	1.00		
Good	1.51	0.191	0.81–2.82
Fair	**2.94**	**0.002**	**1.49–5.79**
Bad/very bad	**2.94**	**0.012**	**1.27–6.81**
**Diabetes duration**			
< 10 years^a^	1.00		
10–19 years	1.23	0.350	0.79–1.94
20–29 years	1.76	0.052	0.99–3.09
30 years and over	**3.24**	**0.022**	**1.19–8.83**

Statistically significant findings are marked in bold

OR odds ratio; CI confidence interval

a: Reference groups

b: Low (primary/part of secondary school), Middle (high school), High (college/university < 4 years), Highest (college/university 4 years or more)

### Associations of education, self-rated health, and diabetes duration

In both regression models, specialist visits were associated with the adjustment independent variables education, self-rated health, and diabetes duration, overall tending to a higher probability of specialist visits with higher education, poorer self-rated health, and a longer diabetes duration (Tables 2 and 3). The probability of visiting specialist services was around the double for people with high education, compared to the low education group. People with fair or bad/very bad health were 2–3 times more likely to visit a specialist clinic, and people with a diabetes duration of 30 years and more were 3–4 times more likely to visit a specialist outpatient clinic, compared to the reference groups (excellent health or diabetes duration < 10 years, respectively).

All findings persisted after introducing the response time variable into the regression models (S1 Table). Likewise, the results persisted after excluding the 21 individuals who had made only one GP visit during the previous year (S2 Table).

We found no strong correlations (defined as Spearman rho >0.5) between the independent variables in any of the models. The highest correlation was found for the variables age and diabetes duration (0.350). Testing by Pearson’s correlation test gave a similar result.

## Discussion

### Key findings

We found a UPC of 0.85 among people with T2D. Mean age was 62.6 years. Two thirds of the sample (66.0%) had made all visits to the regular GP during the previous year (full continuity). Among 256 participants with UPC = 1, 48.1% had made one or more specialist visits during the previous year, compared to 65.2% among those with UPC<1. The probability of outpatient specialist visits was significantly lower among participants with full continuity, compared to those who had made at least one visit to other primary care doctors. The probability of visiting outpatient specialist services was not associated with duration of the patient-GP relationship. The probability of specialist visits was significantly higher with higher education, poorer self-rated health, and a longer diabetes duration.

### Mean Usual Provider Continuity (UPC)

UPC was 0.85 in our sample, meaning that 17 out of 20 GP visits were made to the usual provider. In the general Norwegian population, an overall UPC of 0.78 has been reported [37]. UPC increased with patient age and consultation rates [37]. Our sample had a rate of 5.08 consultations per year, compared 2.52 in the general population [37]. High consultation rates may reflect chronic disease [14]. Our UPC estimate corresponds to what would be expected in a T2D population with a high mean age and a high consultation rate.

A recent Norwegian register-based study found a mean UPC of 0.70 in a diabetes population, including type 1 diabetes (T1D) as well as T2D [39]. Despite the inclusion of T1D, mean age of participants was 63.9 years, which is slightly higher than in our study. Consultation rates in the studies were the same (around 5 per year). The UPC estimate in the register-based study was surprisingly low compared to our study and also compared to UPC in the general population. Since this study included people with both diabetes types, and only if they had a minimum of two diabetes-related consultations during a year (2014), comparisons between the studies are difficult.

Internationally, a study from Canada also reports a UPC of 0.85 in a diabetes population [40]. Another Canadian study found a UPC of 0.84 [24], whereas a US study reports a UPC of 0.59 in a diabetes population [41]. The Canadian health care system is not fundamentally different from the Norwegian, whereas health care in the US differs substantially. From this perspective, it is not surprising that UPC in our study equals the Canadian studies. We have not found studies reporting a higher UPC than 0.85 in diabetes populations. Since continuity is particularly important for persons with chronic disease, our high UPC may indicate that the RGP system with personal lists in Norway to a large extent has reached the ambition of continuity for people with diabetes. In light of the ongoing recruitment crisis in general practice, GPs’ willingness to be available for personal continuity should not be taken for granted in the future.

### Continuity and outpatient specialist visits

Among 256 participants with full continuity (UPC = 1), 48.1% had made one or more specialist visits during the previous year, compared to 65.2% among those without full continuity (UPC<1). The probability of specialist visits was significantly lower among participants with full continuity, compared to those who had made one or more visits to other primary care doctors during the previous year (Table 2). Many studies have reported associations between continuity and reduced overall health service utilization [22], use of emergency departments [23], hospitalizations [22–24], and medical costs [22, 23] in diabetes populations. In particular, there is wide agreement regarding personal continuity and reduced hospitalisations, however, there is little evidence on whether continuity may affect the use of outpatient specialist services in diabetes populations. We do not know which specialist consultations that were avoided in those with UPC = 1 (full continuity). Patients with T2D may of course benefit from specialist visits, however, some may not. Since the literature describes an association between continuity and reduced mortality [12, 19], we have reason to believe that the avoided specialist consultations were mainly those that would not have provided better outcome for the patients. Continuity of GP care may thus be important in terms of ensuring access for those who most likely benefit from specialist care, while preventing unwarranted non-GP specialist visits for those who do not. From clinical experience and previous research, we suggest that the main mechanism for lower specialist use associated with continuity is mutual trust in the patient-GP relationship based on knowing each other for a long time [14].

Even if we found a lower probability of specialist visits among patients with full continuity (UPC = 1), we could not confirm a similar association using duration of the patient-GP relation as measure of continuity. We were surprised by this interesting result. It contrasts our 2013 study, finding that a longer duration of the GP-patient relationship was associated with reduced use of outpatient specialist services [14]. However, the general population studied in 2013 might differ substantially from the current study population with chronic disease. People with T2D are probably more in need of specialist follow-up with increasing age, disease duration, and duration of the patient-GP relationship compared to the general population. A possible difference in associations of this continuity measure between general populations and T2D populations (or other populations with chronic disease), should be subject for further research.

### Education, self-rated health, and diabetes duration

Our finding that specialist visits were associated with higher education, poorer self-rated health, and a longer diabetes duration is not surprising and in line with others’ findings. A large body of research from various health care systems indicates that people with high socio-economic status use outpatient specialist services more than the less advantaged, countries with universal insurance and gate keeping being no exception [42, 43]. Since poorer self-rated health and longer diabetes duration might be seen as need equivalents, it is also not surprising that the probability of using outpatient specialist health services is higher in these groups.

### Strengths and limitations

Particular strengths of this study were the nationwide diagnose specific sample, and the comprehensive coverage of information about health, disease, and socio-economic status in the systematically tested questionnaires. Other strengths were the focus on a scarcely investigated research area, accessing recently collected data (2018).

Even if response rate must not be confused with response quality, a limitation of this study is the low estimated participation rate, one of the indicators of study representativeness [38]. However, assessment of the possible influence of non-participation on exposure, outcome or the relation of interest is of greater significance than the response rate itself. We have thoroughly accounted for this in several previous studies using the same dataset [34, 44–47]. More importantly, our efforts to investigate possible skewness from any possible selection bias in this particular study revealed no significant flaws. Still, assuming that late respondents are more similar to non-respondents younger individuals, men, people in poorer health and people with more specialist visits might have been overrepresented since these groups dominated slightly among the late respondents (Table 1) [38]. Poorer health and more specialist visits might be connected; however, younger age is not likely to be associated with these factors. I addition, participation in surveys is usually lower in younger ages, adding a contribution to balance out a possible overrepresentation of younger individuals in this study [48].

In questionnaire data, there is always a potential for recall bias, usually leading to underreporting of minor and distant events. Likewise, the validity of self-reported data might be questioned, however, agreement between self-reported and registered health and health care utilization is generally considered high [49, 50].

Recruitment from a patient organization means that we have not reached the non-organized. People engaged in a patient organization might be more motivated with a better GP engagement compared to non-members. Thus, it might be questioned whether our data is representative for the T2D population in Norway. Solid national data on sociodemographic and disease related characteristics of people with T2D are lacking, however, there are indices that people with T2D have lower education compared to our sample [51]. Since it is well known that the probability of visiting specialist services increases with higher education [42], our findings of limited use of specialist services with high UPC might be considered even stronger with the relatively high education in our sample in mind. Our sample contains slightly more men than the national average [2], however, since participation in surveys are generally higher in women [48], this might also not affect the validity of our results.

As with all cross-sectional studies, no causal relationships can be established. Finally, we cannot exclude the possibility of unmeasured confounders of the reported associations.

Summing up, we suggest that the mentioned limitations posed a limited threat to our study’s validity, and it is not possible to judge the magnitude of a possible bias, since different factors might level each other out. The low estimated response rate is not in itself an indication of low representativeness, as non-response bias may be present even with high response rates [38]. Nevertheless, generalisation and interpretation must be made with caution.

## Conclusions

We conclude that continuity of care, as measured by Usual Provider Continuity, is high and associated with reduced use of outpatient specialist services in people with type 2 diabetes in Norway. Since limiting unwarranted use of specialist care is a health policy goal in Norway, one may do well to perform and organise health services in ways that support continuity of GP care. Continuity is reduced by the ongoing recruitment crisis in general practise, while its benefits will become increasingly important as the number of older people with diabetes and other chronic diseases increases.

## Supporting information

S1 Questionnaire (PDF)

S1 Table Probability of outpatient specialist visits adjusted for response time. (PDF)

S2 Table Probability of outpatient specialist visits for patients with two or more GP visits during the previous year. (PDF)

## List of abbreviations

NDA: Norwegian Diabetes Association
NSD: The Norwegian Centre for Research Data
REK: Regional Committee for Medical and Health Research Ethics
OR: Odds ratio
CI: Confidence interval
T2D: Type 2 diabetes
