# Supplementary material for: Personal continuity of GP care and outpatient specialist visits in people with type 2 diabetes: A cross-sectional survey
Source: PLoS One. 2022 Oct 25;17(10):e0276054. doi: 10.1371/journal.pone.0276054 (PMC9595526; doi:10.1371/journal.pone.0276054)
Supplement: S1 Table — (PDF) [file pone.0276054.s002.pdf]

S1 Table A. Probability of one or more outpatient specialist visits according to Usual Provider Continuity (UPC) (N=472), additionally adjusted for response time

	Outpatient specialist visits (yes/no)		
	**OR**	**p**	**CI**
**UPC[a]**			
<1[b]	1.00		
1	**0.53**	**0.003**	**0.35-0.80**
**Gender**			
Female[b]	1.00		
Male	1.11	0.622	0.74-1.67
**Age**			
18-39 years[b]	1.00		
40-59 years	0.77	0.699	0.21-2.86
60 years and over	1.17	0.817	0.31-4.36
**Education[c]**			
Low[b]	1.00		
Middle	1.22	0.541	0.65-2.29
High	**1.94**	**0.044**	**1.02-3.69**
Highest	1.72	0.108	0.89-3.33
**Self-rated health**			
Excellent[b]	1.00		
Good	1.50	0.203	0.80-2.82
Fair	**2.80**	**0.003**	**1.41-5.57**
Bad/very bad	**2.75**	**0.020**	**1.18-6.46**
**Diabetes duration**			
< 10 years[b]	1.00		
10-19 years	1.15	0.541	0.73-1.82
20-29 years	1.74	0.058	0.98-3.07
30 years and over	**2.97**	**0.034**	**1.08-8.14**
**Response time[d]**			
Early respondents [b]	1.00		
Late respondents	0.69	0.073	0.46-1.03

Statistically significant findings are marked in bold

OR odds ratio; CI confidence interval

a: UPC Usual Provider Continuity <1: some visits to other than regular provider, UPC 1: all visits to regular provider

b: Reference groups

c: Low (primary/part of secondary school), Middle (high school), High (college/university < 4 years), Highest (college/university 4 years or more)

d: Early respondents (responded initially), Late respondents (responded after one reminder)

S1 Table B. Probability of outpatient specialist visits according to duration of the patient-GP relation (N=472), additionally adjusted for response time

	Outpatient specialist visits (yes/no)		
	**OR**	**p**	**CI**
**Duration of the patient-GP relation**			
0-4 years [a]	1.00		
>4 years	0.95	0.819	0.62-1.46
**Gender**			
Female[a]	1.00		
Male	1.03	0.894	0.69-1.54
**Age**			
18-39 years[a]	1.00		
40-59 years	0.72	0.627	0.19-2.65
60 years and over	1.07	0.915	0.29-3.96
**Education[b]**			
Low[a]	1.00		
Middle	1.30	0.412	0.70-2.43
High	**2.02**	**0.030**	**1.07-3.83**
Highest	1.85	0.064	0.97-3.56
**Self-rated health**			
Excellent[a]	1.00		
Good	1.54	0.173	0.83-2.89
Fair	**2.95**	**0.002**	**1.49-5.84**
Bad/very bad	**2.88**	**0.014**	**1.24-6.70**
**Diabetes duration**			
< 10 years[a]	1.00		
10-19 years	1.23	0.375	0.78-1.93
20-29 years	**1.78**	**0.047**	**1.01-3.14**
30 years and over	**3.20**	**0.023**	**1.17-8.72**
**Response time[d]**			
Early respondents[b]	1.00		
Late respondents	0.70	0.073	0.47-1.03

Statistically significant findings are marked in bold

OR odds ratio; CI confidence interval

a: UPC Usual Provider Continuity <1: some visits to other than regular provider, UPC 1: all visits to regular provider

b: Reference groups

c: Low (primary/part of secondary school), Middle (high school), High (college/university < 4 years), Highest (college/university 4 years or more)

d: Early respondents (responded initially), Late respondents (responded after one reminder)
