# Supplementary material for: Personal continuity of GP care and outpatient specialist visits in people with type 2 diabetes: A cross-sectional survey
Source: PLoS One. 2022 Oct 25;17(10):e0276054. doi: 10.1371/journal.pone.0276054 (PMC9595526; doi:10.1371/journal.pone.0276054)
Supplement: S2 Table — (PDF) [file pone.0276054.s003.pdf]

S3 Table A. Probability of one or more outpatient specialist visits according to Usual Provider Continuity (UPC) (N=453) for patients with two or more GP visits during the previous year

	Outpatient specialist visits (yes/no)		
	**OR**	**p**	**CI**
**UPC[a]**			
<1[b]	1.00		
1	**0.53**	**0.003**	**0.35-0.81**
**Gender**			
Female[b]	1.00		
Male	1.13	0.534	0.75-1.72
**Age**			
18-39 years[b]	1.00		
40-59 years	0.76	0.686	0.21-2.81
60 years and over	1.12	0.862	0.33-4.16
**Education[c]**			
Low[b]	1.00		
Middle	1.20	0.578	0.63-2.31
High	**1.94**	**0.051**	**1.00-3.65**
Highest	1.60	0.174	0.81-3.15
**Self-rated health**			
Excellent[b]	1.00		
Good	1.58	0.159	0.84-2.97
Fair	**2.87**	**0.003**	**1.44-5.70**
Bad/very bad	**3.20**	**0.009**	**1.33-7.67**
**Diabetes duration**			
< 10 years[b]	1.00		
10-19 years	1.04	0.865	0.65-1.66
20-29 years	1.57	0.126	0.88-2.80
30 years and over	**3.07**	**0.041**	**1.04-9.01**

Statistically significant findings are marked in bold

OR odds ratio; CI confidence interval

a: UPC Usual Provider Continuity <1: some visits to other than regular provider, UPC 1: all visits to regular provider

b: Reference groups

c: Low (primary/part of secondary school), Middle (high school), High (college/university < 4 years), Highest (college/university 4 years or more)

reminder)

S3 Table B. Probability of one or more outpatient specialist visits according to duration of the patient-GP relationship (N=453) for patients with two or more GP visits during the previous year

	Outpatient specialist visits (yes/no)		
	**OR**	**p**	**CI**
**Duration of the patient-GP relation**			
0-4 years [a]	1.00		
>4 years	0.94	0.806	0.61-1.47
**Gender**			
Female[a]	1.00		
Male	1.06	0.765	0.71-1.60
**Age**			
18-39 years[a]	1.00		
40-59 years	0.71	0.609	0.20-2.59
60 years and over	1.04	0.954	0.28-3.80
**Education[b]**			
Low[a]	1.00		
Middle	1.30	0.428	0.68-2.47
High	**2.03**	**0.034**	**1.05-3.92**
Highest	1.74	0.104	0.89-3.39
**Self-rated health**			
Excellent[a]	1.00		
Good	1.62	0.131	0.87-3.05
Fair	**3.02**	**0.002**	**1.52-5.99**
Bad/very bad	**3.31**	**0.007**	**1.40-7.87**
**Diabetes duration**			
< 10 years[a]	1.00		
10-19 years	1.11	0.649	0.70-1.76
20-29 years	1.61	0.103	0.91-2.87
30 years and over	**3.36**	**0.027**	**1.15-9.82**

Statistically significant findings are marked in bold

OR odds ratio; CI confidence interval

a: UPC Usual Provider Continuity <1: some visits to other than regular provider, UPC 1: all visits to regular provider

b: Reference groups

c: Low (primary/part of secondary school), Middle (high school), High (college/university < 4 years), Highest (college/university 4 years or more)
